# Transcriptome Analyses Reveal Expression Profiles of Morphologically Undifferentiated and Differentiated Gonads of Yangtze Sturgeon *Acipenser dabryanus*

**DOI:** 10.3390/genes14112058

**Published:** 2023-11-09

**Authors:** Rui Ruan, Ying Li, Huamei Yue, Huan Ye, Jiali Jin, Jinping Wu, Hao Du, Chuangju Li

**Affiliations:** 1 Key Laboratory of Freshwater Biodiversity Conservation, Ministry of Agriculture and Rural Affairs of China, Yangtze River Fisheries Research Institute, Chinese Academy of Fishery Sciences, Wuhan 430223, China; ruanrui@yfi.ac.cn (R.R.); liying5856@163.com (Y.L.); yhuam@yfi.ac.cn (H.Y.); yehuan@yfi.ac.cn (H.Y.); jljin@yfi.ac.cn (J.J.); wujinping@yfi.ac.cn (J.W.); 2 Laboratory of Freshwater Fish Germplasm Resources and Biotechnology, Yangtze River Fisheries Research Institute, Chinese Academy of Fishery Sciences, Wuhan 430223, China

**Keywords:** Yangtze sturgeon, gonadal differentiation, transcriptome analyses, *foxl2*

## Abstract

Sturgeon is known as a primitive fish with the ZZ/ZW sex determination system and is highly prized for its valuable caviar. Exploring the molecular mechanisms underlying gonadal differentiation would contribute to broadening our knowledge on the genetic regulation of sex differentiation of fish, enabling improved artificial breeding and management of sturgeons. However, the mechanisms are still poorly understood in sturgeons. This study aimed to profile expression patterns between female and male gonads at morphologically undifferentiated and early differentiated stages and identify vital genes involved in gonadal sex differentiation of sturgeons. The sexes of Yangtze sturgeon (*Acipenser dabryanus*) juveniles were identified via the sex-specific DNA marker and histological observation. Transcriptome analyses were carried out on female and male gonads at 30, 80 and 180 days post-hatching. The results showed that there was a total of 17 overlapped DEGs in the comparison groups of between female and male gonads at the three developmental stages, in which there were three DEGs related to ovarian steroidogenesis, including *hsd17b1*, *foxl2* and *cyp19a1*. The three DEGs were highly expressed in the female gonads, of which the expression levels were gradually increased with the number of days after hatching. No well-known testis-related genes were found in the overlapped DEGs. Additionally, the expression levels of *hsd17b1* and *cyp19a1* mRNA were decreased with the knockdown of *foxl2* mRNA via siRNA. The results further suggested that *foxl2* should play a crucial role in the ovarian differentiation of sturgeons. In conclusion, this study showed that more genes involved in ovarian development than testis development emerged with sexually dimorphic expression during early gonadal sex differentiation, and it provided a preliminary understanding of the molecular regulation on gonadal differentiation of sturgeons.

## 1. Introduction

The order *Acipenseriformes* (consisting of sturgeons and paddlefishes) is considered to be a primitive group of actinopterygian fish that plays vital roles in fish phylogeny. Sex determination of sturgeons and paddlefishes belongs to the female heterogametic type of genetic determination [1,2]. As yet, little is known of the sex-determining molecular mechanisms in the order *Acipenseriformes*. Elucidating the molecular process during gonadal differentiation of sturgeons would be beneficial by broadening our understanding of their sex differentiation, further uncovering their sex-determining mechanisms.

In contrast to mammals and birds with obvious heterotypic chromosomes, most fish species have no highly distinct and differentiated sex chromosomes. Moreover, different sex determination systems and master sex-determining genes exist in fishes. Medaka fishes of the genus *Oryzias* possess different sex determination systems. For instance, *Oryzias latipes*, *Oryzias curvinotus*, *Oryzias dancena*, *Oryzias minutillus* and *Oryzias luzonensis* have an XX/XY system, whereas *Oryzias hubbsi* and *Oryzias javanicus* have a ZZ/ZW system [3,4]. They also have different master sex-determining genes; for instance, *Dmy* (DM-domain gene on the Y chromosome) has been found to be a master sex-determining gene in *O. latipes* [5] and *O. curvinotus* [6], while the master sex-determining gene in *O. luzonensis* was *Gsdf^Y^* (gonadal-soma-derived growth factor on the Y chromosome), which has replaced *Dmy* in the evolutionary process [7]. Therefore, the mechanism of sex determination in fish is highly diverse and plastic, which also increases the difficulty of exploring the molecular process of sex differentiation.

To investigate key genes involved in sex determination and differentiation in sturgeons, expression patterns of sex-related genes such as *dmrt1*, *sox9*, *foxl2* and *cyp19a1* have been analyzed by other researchers. These genes exhibited sexually dimorphic expression in the ovary and testis at certain developmental stages in their study [8,9,10,11,12,13]. With the rapid development of genomics, transcriptomics and other omics technologies, omics has become one of the effective methods for mining master sex-determining genes and uncovering molecular mechanisms of sex determination and differentiation [14,15,16]. Gonadal transcriptome sequencing was employed to discover more putative sex-related genes in Adriatic sturgeon (*Acipenser naccarii*) [17], Chinese sturgeon (*Acipenser sinensis*) [18,19,20], Russian sturgeon (*Acipenser gueldenstaedtii*) [21,22,23], Yangtze sturgeon (*Acipenser dabryanus*) [24], Siberian sturgeon (*Acipenser baeri*) [25,26], lake sturgeon (*Acipenser fulvescens*) [27,28] and Amur sturgeon (*Acipenser schrenckii*) [29,30,31,32,33]. Whole genomes have been sequenced from the sterlet (*Acipenser ruthenus*) [34,35] and American paddlefish [36]. And, to explore the mechanism of sex determination of sturgeons, whole-genome inter-sex variation was comprehensively analyzed using whole-genome sequencing on DNA from five female and five male adult Russian sturgeon [37]. Nevertheless, the molecular mechanism of sexual determination and differentiation remain unclear in sturgeons.

Nearly non-existent obvious secondary sexual characteristics, long lifespan and late maturation [38,39,40,41] might make it difficult to discover master genes or molecular mechanisms underlying the sex differentiation of sturgeons. At present, sex-specific DNA sequences and markers of sturgeons were obtained by comparative genomics with high-throughput sequencing [2,42], which could contribute to identifying the genetic gender of sturgeons early in development. And, the sex-specific DNA markers have been used to identify the sexes of lake sturgeon (*Acipenser fulvescens*) [43] and Yangtze sturgeon [44]. Morphological sex differentiation of gonads could be observed with histological section, for instance, in Yangtze sturgeon by 78 days post-hatching (dph) [45]. However, vital sex-differentiation-related genes might express differentially prior to morphological sex differentiation of gonads. The previous gonadal transcriptome analyses mainly focused on differentiated gonads, which did not reflect genes involved in the onset of sex differentiation. With the help of sex-specific DNA markers, profiling changes in gene expression during gonadal differentiation would be more feasible to carry out using transcriptome analysis.

In this study, undifferentiated and differentiated gonads of Yangtze sturgeon were collected and examined using sex-specific DNA marker and histological sections. Gene expression profiles among these gonads were investigated using mRNA-Seq. By integrated analyses of these transcriptome profiles, differentially expressed genes (DEGs) during sex differentiation were screened out. This study aimed to provide basic information on the transcriptome level during gonadal differentiation to assist future studies to elucidate the molecular mechanisms of sex determination and differentiation of sturgeons.

## 2. Materials and Methods

### 2.1. Fish and Sampling

Yangtze sturgeon juveniles were reared in 1600-L tanks from Taihu station, Yangtze River Fisheries Research Institute, Chinese Academy of Fisheries Science. A total of 60 juveniles from 30 dph, 70 dph, 80 dph, 90 dph and 180 dph were randomly selected, twelve fishes from each developmental stage. They were anesthetized with 0.001% ethyl 3-aminobenzoic acid ethyl ester methanesulfonate-222 (Sigma, Burlington, MA, USA) and were sacrificed by quick decapitation. The tail fin tissue was collected and kept in ethanol for genomic DNA extraction. Additionally, a piece of gonadal tissue was fixed in Bouin’s solution for histological identification, and the remnant was preserved in RNA Save (Biological Industries, Kibbutz Beit Haemek, Israel) for RNA extraction. These experimental procedures complied with the guiding principles of the Animal Care and Use Committee of the Yangtze River Fisheries Research Institute, Chinese Academy of Fishery Sciences.

### 2.2. Sex Identification Using Sex-Specific DNA Marker

Genomic DNA was extracted from the tail fin tissue of the juveniles using the TIANamp Genomic DNA kit (Tiangen, Beijing, China) according to the manufacturer’s instructions and assessed using a 1.5% agarose gel and a NanoDrop Lite spectrophotometer (Thermo Fisher Scientific, Waltham, MA, USA). A pair of sex-specific primers were synthesized according to the published sequences (5′-TAATCAATTGTAAGTCGCCAAG-3′ and 5′-ATTTTATTACGGTGAGTATACGAA-3′) [2]. A traditional PCR was conducted with the following steps: 95 °C for 5 min; 35 cycles of 95 °C for 30 s, 52 °C for 45 s and 72 °C 45 s; and 72 °C for 7 min. Each amplification reaction contained 1 μL (approximately 100 ng) DNA template, 10.5 μL ddH2O, 0.5 μL each primer (10 μM) and 12.5 μL 2 × Premix Taq (Takara, Shiga, Japan) in a total volume of 25 μL. The PCR products were detected using electrophoresis with 1.5% agarose gel.

### 2.3. Histological Analysis

Gonads fixed in Bouin’s solution overnight were transferred to 70% ethanol at 4 °C until paraffin sectioning. Samples were cut into 4 μm thick slices in a standard paraffin embedding method and stained with hematoxylin-eosin (HE). Images of sections were obtained using a light microscope (BX-51, Olympus, Tokyo, Japan) and a digital camera (DP-73, Olympus).

### 2.4. Transcriptome Sequencing and Analyses

Total RNA was extracted from gonads with RNeasy Plus Mini Kit (Qiagen, Venlo, The Netherlands) according to the manufacturer’s instructions. RNA degradation and concentration were preliminarily assessed by 1.5% agarose gel electrophoresis and Nanodrop Lite spectrophotometer (Thermo Scientific, Waltham, MA, USA). The RNA samples were sent to Beijing Novogene Bioinformatics Technology Co., Ltd. (Beijing, China) for further assessment and library construction. The Illumina HiSeq 4000 platform was used to generate 150 bp paired-end reads. Clean data were obtained by removing low-quality raw reads that contained more than 10% poly-N or 50% bases with Qphred < 5. Due to no reference genome of Yangtze sturgeon, de novo assembly of a Yangtze sturgeon reference transcriptome was carried out using Trinity [46] with min_kmer_cov set to 2 by default and all other parameters set to default. To refine the final transcriptome dataset, a further hierarchical clustering step was performed using Corset [47]. The longest transcript of multiple isoform Corset clusters was selected as representative of the cluster, called ‘unigene’. Function annotation of the unigenes was conducted against the National Center for Biotechnology Information (NCBI) non-redundant protein sequences database (Nr), NCBI nucleotide sequences database (Nt), Swiss-Prot database, Protein families database (Pfam), clusters of orthologous groups for eukaryotic complete genomes database (KOG), Gene Ontology database (GO) and Kyoto Encyclopedia of Genes and Genomes Database (KEGG). Gene expression levels were estimated using RSEM [48] for each sample. Differential expression analyses between different groups were performed using DESeq2 [49], which used a negative binomial distribution to model the RNA-seq counts for determining differential expression in digital genes. The *p*-values from differential expression significance analysis were further adjusted using Benjamini and Hochberg’s approach for controlling the false discovery rate. Genes with an adjusted *p*-value < 0.05 and an absolute value of log2(fold change) > 1 were recognized as significant differential expression.

### 2.5. In Vivo Experiment and RNA Interference

The *foxl2* siRNA was designed using DSIR (http://biodev.extra.cea.fr/DSIR/DSIR.html, accessed on 1 November 2023). The double-strand *foxl2* siRNA (sense 5′-CAUGUGAAGACAUGUUUGAGA-3′, antisense 5′-UCAAACAUGUCUUCACAUGCG-3′) and negative control siRNA (sense 5′-UUCAAUUACAUUCAGGCUUAG-3′, antisense 5′-AAGCCUGAAUGUAAUUGAAUG-3′) were synthesized by AuGCT Biotech Co., Ltd., Beijing, China, respectively. Transfection of siRNA was performed using the reagent of Entranster^TM^ in vivo for RNA (Engreen Biosystem, Auckland, New Zealand) according to the manufacturer’s protocol. The *foxl2* siRNA and negative control siRNA were diluted to 1 μg/μL in endotoxin-free pure water. The transfection reagent was diluted to 50% using normal saline. For in vivo transfection, twenty 3-month-old juvenile Yangtze sturgeon (body weight: 21.7 ± 4.7 g) including fifteen females and five males were selected according to the results of sex identification using sex-specific DNA marker. Equal volumes of siRNA (1 μg/μL) and diluted transfection reagent were mixed together. The mixture with 16.7 μg siRNA was injected into the abdominal cavity of female sturgeon through the ventral side of the body. Each of the experimental groups (normal males, normal females, females treated with negative control siRNA, females treated with *foxl2* siRNA) consisted of five individuals. At 48 h post-injection, the gonads were collected to quantify the relative expression levels of *foxl2*, *cyp19a1a* and *hsd17b1* using qRT-PCR.

### 2.6. Real-Time qPCR

Primers of *foxl2*, *cyp19a1*, *hsd17b1* and *β-actin* for qRT-PCR were designed using Oligo software (version 7), of which the sequences were shown as follows: *foxl2* (5′-CTTCCTTTCCCCACCGCCTT-3′ and 5′-ACTCTGTCCGGCATCTACCAGT-3′), *cyp19a1* (5′-TGCACAACACCCCGAGGTTGA-3′ and 5′-TTCCCTTTCTCACAGTGTAGCCTT-3′), *hsd17b1* (5′-AGTTCCATCTTCCACGGTCAGCTT-3′ and 5′-CCAGTCTGCAGCACGTCGACCC-3′) and *β-actin* (5′-TGACAATGCCGTGCTCGATT-3′ and 5′-CATGGAAGACGAAATTGCCGCACT-3′). The cDNA templates were reverse-transcribed with total RNA from the gonads using the PrimerScript™ RT reagent kit with gDNA Eraser (TaKaRa, Shiga, Japan) following the manufacturer’s instructions. qRT-PCR was performed on a QuantStudio6 Flex Real time PCR system (Thermo Fisher Scientific, Waltham, MA, USA) using the TB Green^®^ Premix Ex Taq™ II (Tli RNaseH Plus; TaKaRa, Shiga, Japan). Each sample was amplified in triplicate with the following steps: 94 °C for 5 min; 40 cycles of 94 °C for 30 s, 58 °C for 15 s and 72 °C for 15 s. The relative expression levels were calculated using the arithmetic formula 2^−ΔΔCt^ method based on the expression level of *β-actin*. One-way analysis of variance (ANOVA) was performed to compare the relative expression levels between two experimental groups. *p*-values < 0.05 were considered statistically significant. Statistical analyses were conducted using SPSS software version 20.0.

## 3. Results

### 3.1. Sex Identification and Histological Observation

**Figure 1:**
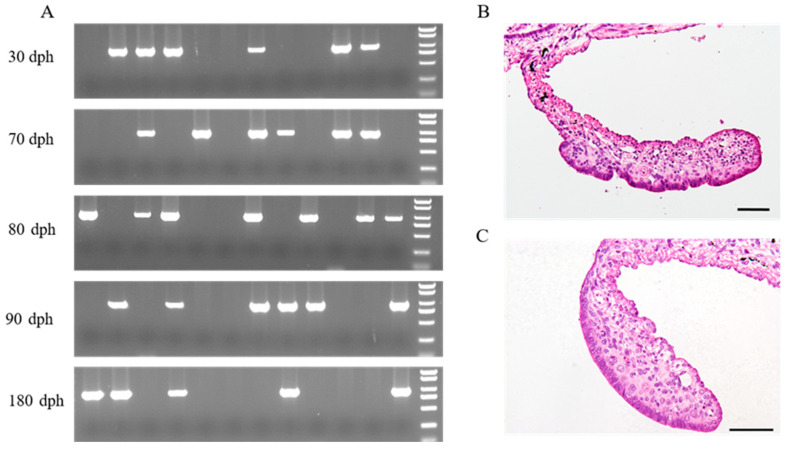
Sex identification of Yangtze sturgeon juveniles and morphological sex differentiation. (**A**) Genetic sex identification of Yangtze sturgeon juveniles using female-specific DNA marker; (**B**) Transverse section of female gonad at 90 days post-hatching (dph); (**C**) Transverse section of male gonad at 90 dph. The rightmost lanes in five agarose gel electrophoresis maps were DL2000 marker DNA (top to bottom: 2000, 1500, 1000, 750, 500, 250 and 100 bp). The sample with a bright band (approximately 900 bp) was identified to be female.

Twelve juveniles from each developmental stage—30 dph, 70 dph, 80 dph, 90 dph and 180 dph—were randomly selected for sex identification and histological observation of the gonads. Based on the results of sex identification using traditional PCR (Figure 1A), five females and five males were chosen from each developmental stage, of which the gonads were histologically examined. The gonads of Yangtze sturgeon at 30 dph, 70 dph and 80 dph did not show evident morphological differentiation between females and males (Figure S1). Morphological gonadal differentiation of female and male Yangtze sturgeon appeared at 90 dph and 180 dph. Female gonads were characterized by a slightly invaginated gonadal epithelium, while male gonads still showed smooth gonadal epithelium (Figure 1 and Figure S1).

### 3.2. Transcriptome Sequencing and Analyses

To explore the gonadal gene expression profiles and vital genes of Yangtze sturgeon in the process of gonadal differentiation, three female and three male gonads during each period (at 30 dph, 80 dph and 180 dph) were selected for transcriptome sequencing. More than 8 Gb (gigabase) of clean data for each sample were obtained for subsequent analyses (Table S1), which were submitted to the NCBI Sequence Read Archive under the accession number PRJNA1022591. In the present study, all the clean data were assembled with the software Trinity, thus generating 698487 transcripts (N50: 930 bp) with a mean length of 627 bp (Table S2). Furthermore, Corset was used to eliminate redundant sequences. The clustered non-redundant transcripts acquired were designated as ‘unigenes’, which included 436558 with an average length of 828 bp and N50 of 1186 bp (Table S2). To obtain comprehensive information on each gene function, unigenes were aligned against seven databases. In total, 217,367 unigenes (49.79%) were annotated in at least one database (Table S3).

**Figure 2:**
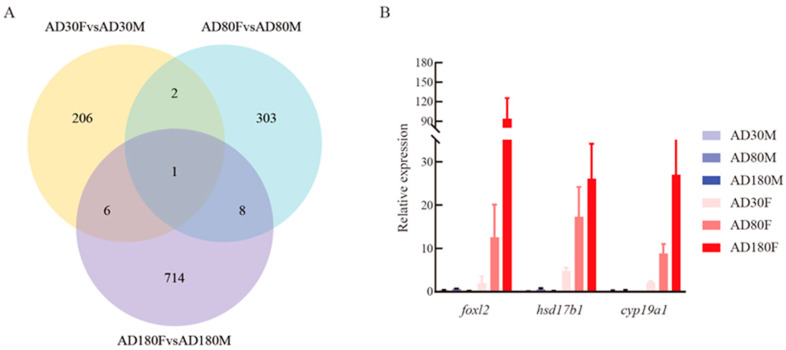
DEGs Venn diagram and validation of vital DEGs using qRT-PCR. (**A**) Venn diagram of DEGs among comparisons of female vs. male gonads at 30 dph, female vs. male gonads at 80 dph and female vs. male gonads at 180 dph. (**B**) The relative expression levels of three overlapped DEGs (*foxl2*, *hsd17b1* and *cyp19a1*) in the three comparison groups were validated using qRT-PCR. AD30F, AD80F and AD180F represent female gonads at 30 dph, 80 dph and 180 dph, respectively; AD30M, AD80M and AD180M represent male gonads at 30 dph, 80 dph and 180 dph, respectively.

The DEGs were identified with the criteria of |log2(fold change)| > 1 and adjusted *p*-value < 0.05 for comparing differential expression patterns between female and male gonads at 30 dph, 80 dph and 180 dph. A total of 215 DEGs were determined from a comparison of female and male gonads at 30 dph. With the increase in the number of days after hatching, the numbers of DEGs in the comparison groups of female vs. male gonads at 80 dph and 180 dph were increased to 314 and 729, respectively. The clustering patterns of DEGs (1258) from each comparison group were analyzed on the basis of their FPKM values (Figure S2). The result indicated that different expression patterns of these DEGs existed among the different comparison groups, which was consistent with the result of the DEGs Venn diagram in the three comparison groups. The Venn plot showed that only one DEG (*hsd17b1*) was shared by the three comparison groups, with a total of 17 overlapped DEGs in the three comparison groups (Figure 2A). In addition, there were eight of the same DEGs between female vs. male gonads at 80 dph and 180 dph, of which two, including *foxl2* and *cyp19a1,* were related to estrogen synthesis on the basis of their functional annotation. According to the FPKM values of *hsd17b1*, *foxl2* and *cyp19a1*, the three DEGs upregulated in female rather than male gonads, the expression level gradually increased in female gonads within the development period (Table S4). In addition, the three DEGs were chosen to verify the reliability of transcriptome data using qRT-PCR. The results of qRT-PCR showed that the expression patterns of the three DEGs were consistent with those obtained from RNA-seq (Figure 2B, Table S5), which showed that the expression analyses based on transcriptome sequencing were reliable.

Because genes known to be involved in testis development were not detected in the overlapped DEGs in the three comparison groups, we chose eleven genes reported as active in male sexual development to search for unigenes annotated in this study and DEGs in different comparison groups. The ten genes considered were: *dmrt1*, *star*, *amh*, *sox9*, *gsdf*, *igf1*, *wt1*, *cyp17a1*, *ar* and *lh* [8,11]. Only *gsdf* was not found in the set of unigenes; the other genes were matched in the unigenes. Unfortunately, they did not differentially express between female and male gonads at three sampling points (Table S4).

**Figure 3:**
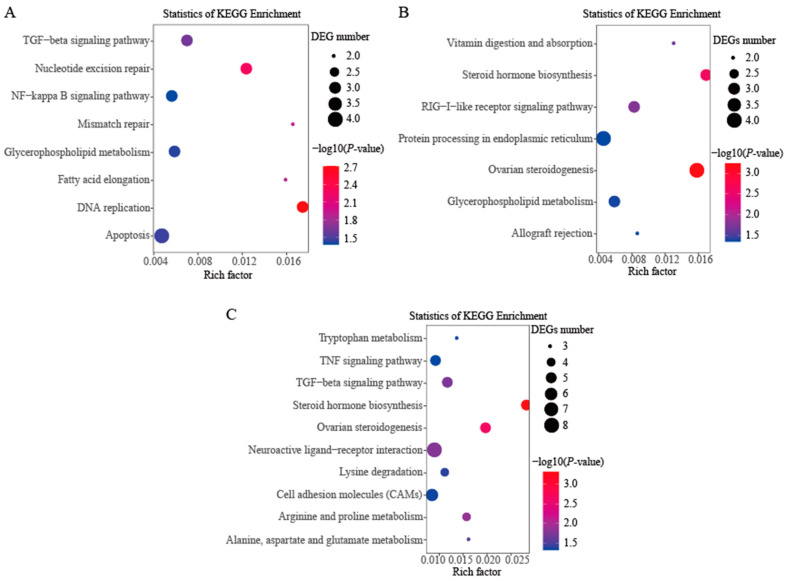
KEGG significantly enriched pathways under no corrected *p*-value among the three comparison groups. (**A**) The comparison group of female vs. male gonads at 30 dph; (**B**) The comparison group of female vs. male gonads at 80 dph; (**C**) The comparison group of female vs. male gonads at 180 dph.

In order to further investigate the DEGs involved in the putative pathways, KEGG pathway enrichment analyses were conducted. No enriched KEGG pathway with a corrected *p*-value less than 0.05 was found among the three comparison groups (Tables S6–S8). In the comparison group of female vs. male gonads at 30 dph, eight pathways—DNA replication, nucleotide excision repair, mismatch repair, fatty acid elongation, TGF-beta signaling pathway, apoptosis, glycerophospholipid metabolism and NF-kappa B signaling pathway (except the class of disease pathways)—were significantly enriched with no corrected *p*-value < 0.05 (Figure 3A). Seven pathways—ovarian steroidogenesis, steroid hormone biosynthesis, RIG-I-like receptor signaling pathway, vitamin digestion and absorption, glycerophospholipid metabolism, protein processing in endoplasmic reticulum and allograft rejection (except the class of disease pathways)—were significantly enriched with no corrected *p*-value < 0.05 in the comparison group of female vs. male gonads at 80 dph (Figure 3B). In the comparison group of female vs. male gonads at 180 dph, we found that ten pathways—steroid hormone biosynthesis, ovarian steroidogenesis, arginine and proline metabolism, neuroactive ligand-receptor interaction, TGF-beta signaling pathway, alanine, aspartate and glutamate metabolism, lysine degradation, tryptophan metabolism, cell adhesion molecules (CAMs) and TNF signaling pathway (except the class of disease pathways)—were significantly enriched with no corrected *p*-value < 0.05 (Figure 3C). Two significantly enriched KEGG pathways—ovarian steroidogenesis and steroid hormone biosynthesis—involved in the process of estrogen biosynthesis were found in two comparison groups, female vs. male gonads at 80 dph and 180 dph.

### 3.3. foxl2 Knockdown In Vivo

**Figure 4:**
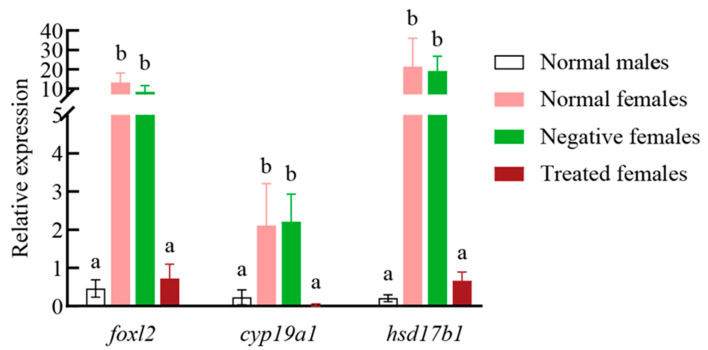
Relative expression levels of *foxl2*, *cyp19a1* and *hsd17b1* in Yangtze sturgeon female gonads treated with *foxl2* siRNA. Normal males: males with no treatment. Normal females: females with no treatment. Negative females: females treated with negative control siRNA. Treated females: Females treated with *foxl2* siRNA. Different lower-case letters indicate significantly different means: *p* < 0.05.

*foxl2* knockdown by siRNA was performed in 3-month-old female Yangtze sturgeon juveniles. The result of qRT-PCR demonstrated that *foxl2* siRNA could significantly reduce the expression of *foxl2* in female gonads (Figure 4). In the female gonads treated with *foxl2* siRNA, the expression levels of *hsd17b1* and *cyp19a1* were checked. Their expression also presented a significant decrease in comparison with control females, while the expression of the three genes showed no difference between normal and negative females (Figure 4, Table S9).

## 4. Discussion

At present, the molecular mechanism of the ZZ/ZW sex determination system remains unclear in vertebrates [50,51,52], especially in fishes. Sturgeon is a primitive fish with the ZZ/ZW sex determination system, with a long lifespan and late puberty (5–30 years of age) [41]. Their preovulatory oocytes are prized as valuable caviar. Investigating expression profiles of sturgeons’ gonads during molecular sex differentiation would be of great benefit for artificial breeding and management and would contribute to our understanding of the mechanism of sex differentiation in fish with the ZZ/ZW sex determination system. In this study, the Yangtze sturgeon was used as a model species of sturgeons to investigate gene expression patterns during gonadal differentiation at the transcriptome level and to discover vital genes involved in gonadal sex differentiation.

To our knowledge, this was the first characterization of the gene expression profiles of undifferentiated and differentiated gonads that occurred in the process of morphological sex differentiation in Yangtze sturgeon juvenile fish with known genetic sex. Morphological sex differentiation of germinal epithelium was observed at 90 dph in this study. Previous studies reported that two distinguishable types of gonads were detected for Yangtze surgeon at 78 dph [45], Russian sturgeon at 3 months post-hatching (mph) [53], Amur sturgeon at 9 mph [10] and shortnose sturgeon (*Acipenser brevirostrum*) at 6 mph [53]. These results showed that there was variation at the beginning of morphological sex differentiation both among different species of sturgeons and within populations of the same sturgeon species.

Generally, molecular changes precede the morphological sex differentiation of gonads in vertebrates [21,54,55]. Since there was no evident external morphological variation between female and male sturgeon, it was difficult to determine the sex of the fish before the onset of morphological gender differentiation by observing external features or examining the sections of gonads. To solve the problem, the genetic gender of Yangtze sturgeon was identified using our previously developed sex-specific DNA marker before the period of morphological gonadal differentiation. Thus, the gene expression profiles of the gonads from female and male Yangtze sturgeon could be determined during the process of gonadal differentiation. In this study, two stages before morphological sex differentiation and one stage after morphological sex differentiation were selected to explore DEGs between female and male gonads. KEGG pathway enrichment analysis was conducted for the DEGs in each comparative group. No KEGG pathway relating to steroidogenesis was significantly enriched in the comparison group of female vs. male gonads at 30 dph. There were two significantly enriched KEGG pathways relative to steroidogenesis, including ovarian steroidogenesis and steroid hormone biosynthesis, in the comparison groups at 80 dph and 180 dph. These results indicated that pathways involved in steroidogenesis had taken part in gonadal development before morphological sex differentiation, which is consistent with the known function of steroidogenesis playing a crucial role in the process of gonadal development including gonadal differentiation, growth and maturation [56].

We found that DEGs in the ovarian steroidogenesis and steroid hormone biosynthesis KEGG pathways were upregulated in female gonads, except one DEG, *cpla2* (Tables S4, S6 and S7). The Venn diagram also showed that three overlapped DEGs (*hsd17b1*, *foxl2* and *cyp19a*) in the three comparison groups were upregulated in female gonads, which were related to female gonadal development. Similarly, the three genes were sexually dimorphic expressions in morphologically differentiated gonads of Russian sturgeon at 9 mph [21] and in morphologically undifferentiated gonads of Siberian sturgeon at 3 mph [57]. To examine the expression level of *foxl2* in gonads of Amur sturgeon during morphological sex differentiation, the results also showed that sexual dimorphic expression of *foxl2* was observed in morphologically differentiated gonads [10]. Similar to most other fish, *foxl2* exhibits a significant sex dimorphic and ovary-dominant expression pattern [55,58,59,60,61,62,63] and is one of the earliest known markers of ovarian differentiation [59]. In a previous work on beluga (*Huso huso*), the results showed that *foxl2* and *cyp19a1* mRNA were sexually dimorphically expressed in female gonads during gonadal sex differentiation and development [12]. The sex-dimorphic expressions in *foxl2* and *cyp19a1* were also validated in 4-year-old Russian sturgeon [23]. *cyp19a1*, which encodes the aromatase-converting androgens to estrogens, plays a critical role in ovarian differentiation, development and growth of teleosts [56]. Additionally, *hsd17b1* is responsible for interconversion between oestrone and oestradiol and between androstenedione and testosterone [64,65]. Since sexually dimorphic expression of the three genes (*hsd17b1*, *foxl2* and *cyp19a*) involved in ovarian steroidogenesis were observed before morphological sex differentiation, the expression levels of which gradually increased with the number of days after hatching, these results suggested that estrogens were indispensable for the ovarian differentiation and development of Yangtze sturgeon.

Though our data showed that *hsd17b1* was the earliest DEG with sex dimorphic expression among the three overlapped DEGs (*hsd17b1*, *foxl2* and *cyp19a*), knockdown of *foxl2* using siRNAs could lead to a decrease in both *hsd17b1* and *cyp19a1* mRNA. *foxl2* regulating cyp19a1 expression is common to all teleost species [56,66]. It is rarely reported that *foxl2* could affect the expression level of *hsd17b1* in fish. The result further suggested that *foxl2* is critical for ovarian differentiation of Yangtze sturgeon as it could affect *hsd17b1* and *cyp19a1* expression. Meanwhile, *foxl2*, except for maintaining *cyp19a1* expression, might play a role during ovarian differentiation by regulating *hsd17b1* expression in sturgeons. Unfortunately, the three genes do not locate on the sex-variable region of Russian sturgeon [37].

Unlike in female gonads of Yangtze sturgeon, it was surprising that no testis-related DEGs were found at the early development stage of Yangtze sturgeon with the ZZ/ZW sex determination system in this study. It is generally believed that males do not rely on testosterone for testicular determination, yet they ask sex-determining or testis-related genes for the onset of testicular differentiation [56]. However, we did not find DEGs related to testicular determination, for instance, *dmrt1* and *amh*. In previous studies on sturgeons, sexual dimorphic expression of *dmrt1* (*dmrt1a* and *dmrt1b*) in gonads was not observed in Amur sturgeon [10] or Russian sturgeon [21] during morphological sex differentiation, but *dmrt1* presenting testis-dominant expression was found in 2-year-old sterlet [11], 3-year-old Siberian sturgeon [9], 3- and 4-year-old Chinese sturgeon [67] and adult Amur sturgeon [10]. It seems that *dmrt1* is not required for early gonadal differentiation of sturgeons, yet it is critical for testis development and growth.

This study provided a snapshot of gene expression profiles during gonadal differentiation at the transcriptome level in Yangtze sturgeon. The results showed that *foxl2*, *cyp19a1* and *hsd17b1* appeared to be involved in the onset of ovarian differentiation, as their sex-dimorphic expression was clearly observed in undifferentiated and differentiated morphological gonads. In future, identifying how *foxl2* affects expression levels of *cyp19a1* and *hsd17b1* would lead to a greater understanding of the onset of ovarian steroidogenesis during sexual development in sturgeons, a primitive fish species. Additionally, research is necessary to investigate expression profiles of the onset of testicular differentiation and development, which would enhance our understanding of the molecular mechanisms of sex differentiation in sturgeons.

## Data Availability

The clean data from gonadal transcriptome sequencing of Yangtze sturgeon juveniles are available from the NCBI Sequence Read Archive under accession number PRJNA1022591.

## Supplementary Materials

The following supporting information can be downloaded at: https://www.mdpi.com/article/10.3390/genes14112058/s1, Figure S1: Transverse section of female and male gonads at four developmental stages including 30 dph, 70 dph, 80 dph, 90 dph and 180 dph; Figure S2: Cluster analysis of DEGs in the three comparison groups. AD30F represents the group of female gonads at 30 days post hatching (dph); AD30M represents the group of male gonads at 30 dph; AD80F represents the group of female gonads at 80 dph; AD80M represents the group of male gonads at 80 dph; AD180F represents the group of female gonads at 180 dph; AD180M represents the group of male gonads at 180 dph; Table S1: Information of high-throughput sequencing data from gonads at the three developmental stages; Table S2: Statistics of the de novo assembly of all clean data; Table S3: Summary of unigenes annotated in seven public databases; Table S4: Differentially expressed genes between female and male gonads at the three developmental stages; Table S5. Raw qRT-PCR data of *foxl2*, *cyp19a1* and *hsd17b1* in the three comparison groups including female vs. male gonads at 30 dph, female vs. male gonads at 80 dph and female vs. male gonads at 180 dph; Table S6: KEGG pathway enrichment analysis of DEGs between female and male gonads at 30 dph; Table S7: KEGG pathway enrichment analysis of DEGs between female and male gonads at 80 dph; Table S8: KEGG pathway enrichment analysis of DEGs between female and male gonads at 180 dph; Table S9. Raw qRT-PCR data of *foxl2*, *cyp19a1* and *hsd17b1* in Yangtze sturgeon female gonads treated with *foxl2* siRNA.
